# Financial Management Behavior Among Young Adults: The Role of Need for Cognitive Closure in a Three-Wave Moderated Mediation Model

**DOI:** 10.3389/fpsyg.2018.02419

**Published:** 2018-11-30

**Authors:** Gabriela Topa, Montserrat Hernández-Solís, Salvatore Zappalà

**Affiliations:** ^1^Department of Social and Organizational Psychology, Universidad Nacional de Educación a Distancia, Madrid, Spain; ^2^Department of Business Economics and Accounting, Universidad Nacional de Educación a Distancia, Madrid, Spain; ^3^Department of Psychology, University of Bologna, Bologna, Italy

**Keywords:** financial management behavior, investment literacy, investment advice use, need for cognitive closure, retirement, retirement planning

## Abstract

This three-wave study aims to explore whether the impact of investment literacy on the financial management behavior is mediated by investment advice use and moderated by the need for cognitive closure. A total number of 272 financially independent adults, under 40 years, completed questionnaires at three different times with 3-month intervals. The results reveal that employees with more investment advice use and characterized by high need for cognitive closure show a higher level of financial management behavior, in relation to both the urgency (seizing) of getting knowledge and the permanence (freezing) of such knowledge. The present study contributes to better understand how and when investment literacy drives well-informed and responsible financial behavior. According to these results, interventions to improve financial behavior should focus on the combination of investment advice use and metacognitive strategies used by individuals to make financial decisions.

## Introduction

Why are some people more efficient in their financial behaviors than others? Financial management is a complex set of behaviors and decisions that can change as a function of the importance and difficulty of implementing the behavior, as well as of people’s capabilities, skills, and opportunities to perform such behaviors. The undesirable short-, mid-, and long-term consequences of inadequate financial management behavior not only affect individuals, but also their household, and ultimately could produce a wide range of unwanted events on the entire society (Fenton et al., 2016). For instance, inadequate financial behaviors can lead to temporary or chronic debts, inability to pay utility bills or filing for bankruptcy and such behaviors result from economic factors together with psychological ones.

Financial literacy has been defined as “the ability and confidence to use one’s own financial knowledge to make financial decisions” (Huston, 2010, p. 307). This concept not only concerns individual investors but also professional ones working in companies that manage money. It is in fact important not only to establish a long-term financial plan but also to know, and to have, financial alternatives in which to invest money or to save it. Financial planning is a very important knowledge and skill considering that individuals live longer and have to save for their old age, when they are no longer working.

Recent studies investigated the impact of financial literacy on various financial behaviors, like loans, mortgages, or retirement planning. The fact that financial literacy is rather low, even across well developed countries, is a critical factor toward well-informed financial decision making and behaviors. Hence, financial behavior management is a topic of interest to economists, social workers and policy makers as well.

However, a large-scale analysis of recent data indicated that financial education interventions explain only 0.1% of the variance in financial behaviors. In contrast, financial literacy has a stronger effect on financial behavior when the former is measured rather than manipulated (Fernandes et al., 2014). However, Fernandes et al. (2014) study shows also that financial literacy has less impact on financial behavior when psychological and social variables, often omitted in previous research, are considered. Therefore, this study aims to fill this gap by taking a psychosocial approach and including cognitive, motivational and social factors in the relationship between financial literacy and financial behavior.

Huston (2010) distinguishes two concepts often considered as synonymous: financial literacy and financial knowledge. A successful measure of financial literacy should allow to identify which outcomes are most impacted by a lack of financial knowledge and skill, and, consequently, allow educators to provide knowledge achieve a desired outcome (Huston, 2010).

In addition, as most of the studies have used samples of students, that is, adolescents or people who are still in their early youth, and not yet financially independent, in this study, we will analyze the financial management behavior of young adults who have their own economic income. Economic independence is in fact a key indicator of transition to adulthood (Lee and Mortimer, 2009).

Based on Huston (2010) theoretical model, this work aims to explore predictors, mediators, and moderators of financial management behavior when people have independent economic resources to save for the future. Specifically, in the present study, we argue that it is necessary to consider the mediating role of investment advice use in the relation between investment literacy and financial management behavior among young adults. As Huston (2010, p. 307) stated, “financial literacy is a component of human capital that can be used in financial activities” to increase behaviors that enhance financial wellbeing. Hence, financial knowledge would be translated in behaviors by using available resources “directly related to successfully navigating personal finances” (Huston, 2010, p. 307), as professional investment advisory services. In addition, we propose that need for cognitive closure (hereafter, NCC), an individual dispositional characteristic, moderates the relations between investment advice use and financial management behavior. The moderated mediation analysis that includes both processes will allow us to better understand the variables that facilitate or hinder young adults’ financial management behavior.

In summary, this study makes three main theoretical and methodological contributions. First, we investigate if the strong direct relationship between financial literacy and financial behaviors is valid when considering two psycho-social variables that consider conditions and types of individuals showing the financial behaviors. Second, we consider younger adulthood, which is a period of individuals’ life-cycle in which many important financial choices start to be made, like buying commodities, a house or setting up a family (Webley et al., 2002). Three, considering what reported by Fernandes et al. (2014), we investigate if the consistent association between financial literacy and financial behavior observed in many cross-sectional studies is observed also when such independent and dependent variables are measured in different moments.

### Financial Management Behavior

Financial management behavior is the acquisition, allocation, and use of financial resources oriented toward some goal. Empirical evidence supports that, if families achieve effective financial management, both their economic well-being and their financial satisfaction improve at the long term (Consumer Financial Protection Bureau, 2015). However, financial management behavior is complex and difficult to implement. The supervision of money and expenditure, which includes frugal and careful spending of money, is a useful protection against risky financial practices.

Moreover, financial management behavior may vary between younger and older people. Although the repeated experience and practice of financial activities influence people’s skills to manage their finances, empirical evidence seems to support that young people practice fewer basic financial tasks, such as budgeting or regularly planning their long-term savings (Jorgensen and Savla, 2010). Because of this evidence, it is of interest to analyze the antecedents of young adults’ financial management behavior.

### Investment Literacy

Investment literacy implies, firstly, an accumulation of knowledge about personal concepts and financial products, obtained by means of education or direct experience. Secondly, it includes a series of abilities and self-confidence to effectively apply the knowledge to the management of one’s own finances. Different empirical works have shown the consistent relations between the specific financial knowledge, the probability of saving, the effectiveness of investment strategies, and saving behaviors in general (Jorgensen and Savla, 2010). Hence, considering we measured our variables at three points in time, we propose that:

*Hypothesis 1:* Investment literacy at time 1 (hereafter T1) will be positively related to financial management behavior at time 3 (hereafter T3).

### Investment Advice Use

The use of financial consultants has been proposed as a useful support to financial decisions and as a substitute of financial knowledge and capacity for individuals and family with lower resources. However, Collins (2012) shows that financial literacy, and search and use of professional advice, are not only distinct and complementary processes, but also positively related, because results show that individuals with higher incomes, better educated and with more financial literacy are the most likely to search and use financial advice. Individuals that are less knowledgeable tend to overestimate their abilities and are unable to recognize their limited financial competences (Kruger and Dunning, 1999). However, other studies show that the use of financial consultants seems to have a direct influence in guiding individuals and families toward more profitable investments (Joo and Grable, 2004). In the light of this evidence, we argue that individuals financially competent, aware of the complexities of the economic field, may search for, understand and then implement the advices provided by financial consultants and, consequently, show good financial management behaviors. Accordingly, we propose that:

*Hypothesis 2:* Investment advice use at time 2 (hereafter T2) will mediate the relationship between investment literacy at T1 and financial management behavior at T3.

### Need for Cognitive Closure

Although some empirical studies have addressed the influence of personality on earning and saving, most of them have focused on psychological biases, self-control problems, procrastination (Rahimi et al., 2016), future time perspective and risk tolerance (Pak and Mahmood, 2015). However, other studies have called attention to the influence of relatively stable individual differences in information processing and complex decision making, such as the NCC (Webster and Kruglanski, 1994).

Need for cognitive closure refers to the individual necessity of arriving to a clear and definitive opinion, or answer to a problem, and particularly any opinion or answer rather than experiencing confusion, ambiguity or inconsistency (Webster and Kruglanski, 1994). Empirical research reports significant differences between people with high and low NCC; such differences concern the amount of information they can process, the intensity of that information, the rules employed in decision-making processes, and the self-confidence on the decisions that they reached (De Dreu et al., 1999; Szumowska and Kossowska, 2017). Due to this characteristic, people with low NCC are more available to consider complex information that is difficult to process, such as financial information. They are also concerned about the loss of information and more oriented toward the accuracy of the response than to the speed with which it is reached. As a consequence, these people tend to consider more information and decide more slowly, to be more open minded and more creative. In contrast, people with high NCC are more likely to focus on information they can process easily, to reject the more complex or even incomplete one (Livi et al., 2015), and less likely to consider new evidence and update their investments when changes in market uncertainty appear (Disatnik and Steinhart, 2015).

Need for cognitive closure has been described as characterized by two different tendencies: the tendency of the urgency to achieve knowledge (*Seizing*) and the tendency to retain permanently that knowledge (*Freezing*) (Roets et al., 2006). People with high NCC have a pressing desire to achieve closure and to retain it permanently. Thus, these people tend to limit the quantity of information to be processed in order to facilitate decision-making and then to retain and perpetuate the information on which they have based this judgment.

This pattern of information processing has been shown in a broad array of situations related to information processing and decision-making (Dolinski et al., 2016), such as consumer purchasing choices, attitudes about complex technological products, suppliers’ purchasing decisions to manage business supply chains, or helping behavior, among others. Due to the fact that financial management behavior includes processing of complex information and the anticipation of needs with a high degree of uncertainty, we argue that individuals with high NCC will consider a limited amount of information provided by the financial consultant, and particularly information that solve their immediate needs; will revise or modify such information with some reluctance, and all this will result in a less efficient financial management behavior. In contrast, we expect that low NCC remain open to information provided by the consultant and, through the elaboration, integration and revision of such information, they will be more consistent and efficient in the management of their financial behavior. Accordingly, in the present study, we propose that:

*Hypothesis* 3: The relationship between investment literacy at T1 and financial management behavior at T3, mediated by investment advice use at T2, will be moderated by both NCC dimensions (seizing and freezing) at T1. Specifically, we expect the relationship between investment advice use (T2) and financial management behavior (T3) to be weaker for individuals with high levels of both NCC dimensions (T1) than for individuals with low levels of both NCC dimensions (T1).

## Materials and Methods

### Ethics Statement

The Institutional Ethics Committee of the first and second authors’ university (National Distance Education University, UNED) approved this research on May 4th, 2016.

### Participants and Procedure

This study, with a three-wave design, was carried out with a sample of young, non-student, Spanish adults, who completed the questionnaires at three different moments (T1, T2, and T3), with an interval of 3 months between each one. Following Taris and Kompier (2016) suggestions, and due to the limited longitudinal studies available on these factors, the real time lag between these factors is unknown; considering literature and the processes under examination, we retain the 3 months as an appropriate period to explore such relations. Also, because the time-lag design contributes to control and counteract the common method variance (Podsakoff et al., 2003). The T1 measurement was carried out in January–February. Participation in the study was voluntarily, and potential participants were informed about the anonymity, and all subjects gave their informed consent for inclusion before they participated in the study. The only inclusion criteria in the study were being younger than 40 years of age and having a paid job (being full time or part time active workers). A total 500 people were invited to participate at T1, but we only obtained 390 responses (78% response rate), and 304 responses at T2. At T3, the sample was reduced to 272 respondents, who are included in this study. The mean age of the participants at T1 was 26.3 years (*SD* = 4.9), and at T3 mean age was 26.8 years. Men made up 40.4% of the sample. Average job seniority was 9.9 years (*SD* = 6.6). In terms of educational level, 57% of the sample had received a university or similar level of education, 29% finished the Secondary School, and 11% had received only basic education. Professionally, 63.2% of participants were employees, 22.8% were middle managers, and full-time workers accounted for 91.9% of the sample, and the rest were employed part-time.

### Instruments

#### Financial Management Behavior

Financial management behavior was assessed with the *Financial Practices Scale* (Loibl et al., 2006), consisting of seven items that measure the probability of the participants’ adopting positive practices of financial management behaviors. The Likert-type response scale ranged from 1 (*unlikely*) to 5 (*very likely*). Examples of some items are: “Pay your bills on time every month”; “Start saving for emergencies”; “Develop a written plan for expenses”; “Have more organized records of payments.” The authors recommend adding the scores to create a global measure of financial management behavior. Reliability was α = 0.78 in the present study.

#### Investment Literacy

Investment literacy was appraised with the *Financial Knowledge Scale*, of Joo and Grable (2004). This 10-item scale was designed to assess investors’ financial literacy. Higher scores indicate more knowledge. The original dichotomic response scale was transformed into a Likert-type response scale ranging between 1 (*strongly disagree*) and 5 (*strongly agree*). Examples of some items are: “Both employee and employer contribute to Social Security”; “Over a 20-year period, one is more likely to win than to lose money in the stock market”; “Interest paid on a credit card is deducted from taxes” (reversed score). Reliability was α = 0.81 in the present study.

#### Investment Advice Use

Investment advice use was assessed using the *Investment Advice Use Scale* of Li et al. (2002) which contains eight items. The original four-point response scale, which ranges between 1 (*strongly disagree*) and 4 (*strongly agree*), was adapted to a five-point Likert-type format, with an intermediate rating for indifference (*neither disagree nor agree*). Examples of items are: “I prefer to consult with a specialist when I take financial decisions”; “I would be willing to pay for the advice of a financial expert”; “I feel qualified to make my own investment decisions without advisors” (reversed score). Reliability was α = 0.77 in the present study.

#### Need for Cognitive Closure

Need for cognitive closure was assessed with the *Need for Cognitive Closure Scale*, in its translated version (Mannetti et al., 2002), adapted to Spanish by Ramelli (2011). This scale has two factors: *Seizing* (predisposition to seek an immediate response when faced with uncertainty) and *Freezing* (predisposition to retain closure and avoid considering new information that might question it). The scale has 14 items that are rated with scores ranging between 1 (*strongly disagree*) and 5 (*strongly agree*). Reliability of the Spanish version was adequate, both in the original study (with α = 0.78; Ramelli, 2011), and in the present study (with α = 0.78). Examples of seizing (urgency) items are: “In case of uncertainty, I prefer to decide immediately, whatever it may be”; “When I have several potentially valid alternatives, I decide in favor of one quickly and without hesitation”; “After finding the solution to a problem, I think it is a waste of time to take other possible solutions into account.” Item examples of the freezing (permanence) dimension are: “I feel very uncomfortable when things are not in their proper place”; “I feel uncomfortable when I do not get a fast answer to a problem I face.” The NCC scale was subjected to Confirmatory Factor Analysis with Amos 24.0. The generalized least squares procedure was used. This two-factor CFA fitted the data reasonably well (χ^2^= 139.199, *p* < 0.000; *df* = 71, CMIN/df = 1.96; GFI = 0.93; AGFI = 0.90, RMSEA = 0.06).

All the factor loadings for the items exceed the 0.40 and both factor correlated as expected (0.72). Some covariances among error have been allowed due to the similarity of the item content, but in any case, between items included under the same factor. Factor loadings, and the Spanish formulation of items, are displayed in Table 1.

**Table 1:** Need of Cognitive Closure Scale (Ramelli, 2011) and factor loadings.

	Factor	
	Freezing	Seizing
En caso de incertidumbre, prefiero tomar una decisión inmediata, sea la que sea (Seizing 1)		0.62
Cuando me encuentro frente a varias alternativas potencialmente válidas, me decido a favor de una rápidamente y sin vacilaciones (Seizing 2)		0.69
Prefiero decidirme de acuerdo con la primera solución disponible, en vez de considerar en detalle qué decisión debería tomar (Seizing 3)		0.66
Cuando necesito enfrentarme a un problema, no pienso mucho sobre él y me decido sin dudar (Seizing 4)		0.52
Cuando necesito solucionar un problema, generalmente no pierdo el tiempo considerando diversos puntos de vista sobre el mismo (Seizing 5).		0.61
Generalmente, no busco soluciones alternativas a problemas para los que ya tengo una solución disponible (Seizing 6)		0.52
Después de encontrar la solución a un problema, considero que es una inútil pérdida de tiempo tener en cuenta otras soluciones posibles (Seizing 7).		0.48
Me siento muy incómodo cuando las cosas a mi alrededor no están en su sitio (Freezing 1).	0.58	
Generalmente, evito participar en discusiones sobre temas ambiguos y controvertidos (Freezing 2)	0.42	
Prefiero estar con personas que tienen las mismas ideas y los mismos gustos que yo (Freezing 3)	0.42	
Me siento incómodo cuando no logro dar una respuesta rápida a un problema al que me enfrento (Freezing 4)	0.77	
Cualquier solución a un problema es mejor que permanecer en un estado de incertidumbre (Freezing 5)	0.53	
Prefiero actividades en las que está siempre claro qué es lo que hay que hacer y cómo hay que hacerlo (Freezing 6)	0.45	
Prefiero cosas a las que estoy acostumbrado que aquéllas que no conozco y no puedo predecir (Freezing 7)	0.44	

### Analytic Strategy

In order to test the study hypotheses, we performed a linear regression analysis. Before testing the hypothesized moderated mediation model, the indirect and moderating effects were first tested separately with the PROCESS macros for SPSS 24 (Hayes, 2013). With bootstrap procedures of 5,000 samples at a 95% confidence level, the confidence intervals that do not contain 0 indicate that the indirect effect is significant. We did not include any control variables in the following analyses.

## Results

Descriptive statistics and Pearson correlations between the study variables are provided in Table 2. Investment literacy was positively and significantly associated both with investment advice use (*r* = 0.19) and with financial management behavior (*r* = 0.31), whereas investment advice use and financial management behavior showed the strongest correlation (*r* = 0.41). The relation between freezing and financial management behavior reached statistical significance (*r* = 0.16). NCC dimensions showed a positive relationship with each other (*r* = 0.44).

**Table 2:** Descriptive statistics and correlation matrix.

*Variables*	*M*	*SD*	*1*	*2*	*3*	*4*
*Predictors*						
1. Investment literacy (T1)	3.2	0.81				
2. Investment advice use (T2)	2.7	0.85	0.19**			
*Outcome*						
3. Financial management behavior (T3)	3.3	0.55	0.31**	0.41**		
*Moderator variables*						
4. NCC-Seizing (T1)	2.7	0.64	-0.11	-0.10	-0.06	
5. NCC-Freezing (T1)	3.3	0.63	0.10	0.06	0.16**	0.44**

*N = 272; *M,* Mean; *SD,* standard deviation; T1, time 1; T2, time 2; T3, time 3. ^∗^*p* < 0.05; ^∗∗^*p* < 0.01*.

Table 3 shows the results obtained when testing the first hypothesis. The linear regression analysis shows the total effect (*b* = 0.17, *p* < 0.000) of investment literacy on financial management behavior [*R*^2^= 0.22, *F*(2,269) = 37.54, *p* < 0.001].

**Table 3:** Regression results of testing the mediation of investment advice use (T2) in the relationships between investment literacy (T1) and financial management behavior (T3) (hypotheses 1 and 2).

Outcome: Investment advice use (T2)	*b*^a^	*SE*	*t*	LLCI 95%	ULCI 95%
Investment literacy (T1)	0.20**	0.06	3.21	0.07	0.32
*R*^2^	0.04*				
*F*_(1,270)_	10.32*				

**Outcome: Financial management behavior (T3)**	***b*^a^**	***SE***	***t***	**LLCI 95%**	**ULCI 95%**

Total effect investment literacy (T1) on financial management behavior (T3)	0.17**	0.03	4.4	0.08	0.23
Direct effect: Investment literacy (T1)→ financial management behavior (T3)	0.16**	0.03	4.3	0.09	0.23
Direct effect: Investment advice use (T2)→ financial management behavior (T3)	0.23**	0.03	6.49	0.16	0.30
Indirect effect: Investment literacy (T1)→ investment advice use (T2)→ financial management behavior (T3)	0.05	0.01		0.01	0.09
*R*^2^	0.22*				
*F*_(2,269)_	37.54*				

*N = 272. ^*a*^ Unstandardized *b* coefficients*; SE,* standard error; CI, confidence interval. Bootstrap = 5,000 samples. ^∗^*p* < 0.01, ^∗∗^*p* < 0.001*.

Regarding the mediation of investment advice use in the relationship between investment literacy and financial management behavior, a significant and positive association between investment literacy and investment advice use (*b* = 0.20, *p* < 0.000) was observed. Furthermore, a statistically significant direct effect of investment literacy on financial management behavior (*b* = 0.16, *p* < 0.001) was found, as well as a statistical significant effect of investment advice use on financial management behavior (*b* = 0.23, *p* < 0.001). Hence, there is a significant indirect effect of investment literacy on financial management behavior through investment advice use (*b* = 0.05). Finally, we tested the significance of this mediation effect through the bootstrapping procedure, which showed that the confidence interval for the indirect effect does not contain zero [0.01, 0.09], supporting the significance of the mediation effect. These results provide reasonable confirmation of hypothesis 2.

Finally, we tested hypothesis 3 following the procedures recommended by Hayes (2013), as shown in Table 4.

**Table 4:** Results of testing the moderation of NCC (T1) on the investment advice use (T2) – financial management behavior relationship (T3) (hypothesis 3).

Criterion variable: Financial management behavior (T3)				

Predictor variable	*b*^a^	*SE*	*t*	LLCI	ULCI
Investment advice use (T2)	0.17	0.04	4.51***	0.09	0.24
NCC-Seizing (T1)	-0.32	0.13	-2.47*	-0.58	-0.07
Interaction investment advice use (T2) × NCC-Seizing (T1)	0.12	0.04	-2.64**	0.03	0.21
*R*^2^	0.24				
*F*_(4,267)_	20.9***				

Investment advice use (T2)	0.15	0.04	4. 21***	0.07	0.22
NCC-Freezing (T1)	-0.21	0.12	-1.62	-0.45	0.04
Interaction investment advice use (T2) × NCC-Freezing (T1)	0.12	0.04	2.58*	0.03	0.21
*R*^2^	0.25				
*F*_(4,267)_	22.3***				

*N = 272. ^*a*^ Unstandardized *b* coefficients; *SE,* standard error; CI, confidence interval. Bootstrap = 5,000 samples. *^∗^p* < 0.05, *^∗∗^p* < 0.01, *^∗∗∗^p* < 0.001*.

Firstly, Table 4 shows a negative direct effect between NCC – seizing and financial behavior (*b* = -0.32, *p* < 0.05), which suggests that the higher the tendency to seek an immediate solution to solve an uncertainty, the lower the management of financial behavior. Secondly, upon testing hypothesis 3 regarding the moderating effect of seizing on the relationship between investment literacy and financial management behavior, mediated by investment advice use, we found a statistically significant positive interaction effect (*b* = 0.12, *p* < 0.01). Thirdly, regarding the moderating effect of freezing on the relationship between investment literacy and financial management behavior, mediated by investment advice use, we also found a statistically significant positive interaction effect (*b* = 0.12, *p* < 0.01). The index of moderated mediation for the seizing dimension was 0.024 (*SE* = 0.013), while the 95% confidence interval with bootstrapping of 5,000 samples did not contain zero (Boot CI [0.003, 0.059]), and for the freezing dimension, the index was 0.023 (*SE* = 0.013, Boot CI [0.002, 0.059]).

Hence, the data support hypothesis 3. The indirect conditional effects of investment literacy on financial management behaviors at the two levels of the moderators are displayed in Table 5, where the effect of investment literacy on financial management behavior was strong at the high level of NCC (seizing and freezing), and it was correspondingly weak when NCC was low. The two effects are statistically significant although in the opposite direction that was expected.

**Table 5:** Results of testing moderated mediation of NCC dimensions in the relationship between investment literacy (T1) and financial management behavior (T3).

	Moderator levels	Conditional effect^a^	Boot *SE*	95% CI	
				Boot LLCI	Boot ULCI
Seizing (T1)	2.01 (Low)	0.03	0.01	0.006	0.07
	3.3 (High)	0.06	0.02	0.01	0.10
Freezing (T1)	2.62 (Low)	0.02	0.01	0.005	0.07
	3.9 (High)	0.06	0.02	0.02	0.11

*N = 272. ^*a*^ Unstandardized *b* coefficients; *SE*, standard error; CI, confidence interval; LL, lower limit; UL, upper limit. Bootstrap = 5,000 samples*.

**FIGURE 1:**
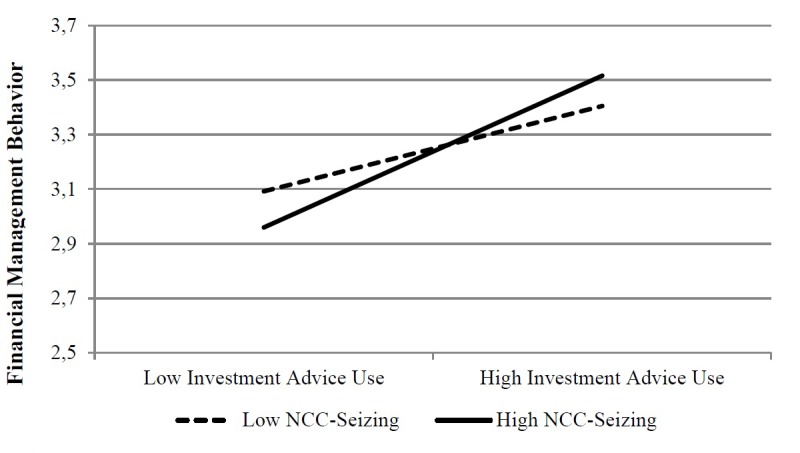
Moderation of NCC-Seizing (T1) on the investment advice use (T2) – financial management behavior (T3) relationship.

**FIGURE 2:**
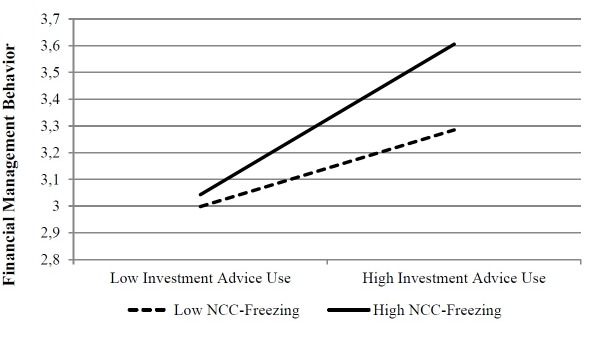
Moderation of NCC-Freezing (T1) on the investment advice use (T2) – financial management behavior (T3) relationship.

Figures 1, 2 depict the moderation effect of both NCC dimensions. What they show is not consistent with our expectations: individuals reporting higher investment advice use also showed a greater level of financial management behavior if they were characterized by high NCC-seizing at T1 (see Figure 1).

Also, contrary to our expectations, respondents reporting higher investment advice use at T2 showed a greater level of financial management behavior at T3 if they were characterized by high NCC freezing at T1 (see Figure 2).

**FIGURE 3:**
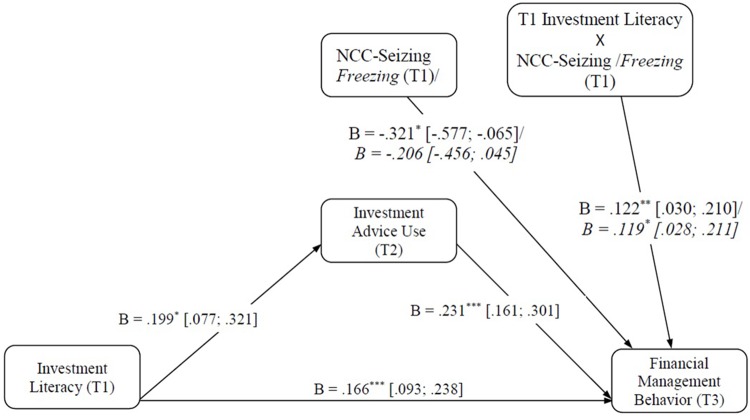
Results of the moderated mediation analysis. NCC, need for cognitive closure; [95% CI]; ^∗^*p* < 0.05, ^∗∗^*p* < 0.01, ^∗∗∗^*p* < 0.001. Values in italics: correspond to the Freezing dimension.

Taken together, this result implies that investment advice use (T2) mediates more strongly the relationship between investment literacy (T1) and financial management behavior (T3) for young adults characterized by moderate to high levels of NCC (T1) than in adults with lower levels of NCC (T1). These results are depicted in Figure 3.

## Discussion

The present work supports the hypothesis that investment literacy may affect subsequent financial management behavior in young, financially independent, adults. These findings corroborate the key assumption of a long research tradition that links financial literacy with the improvement of financial management behavior. In addition, the present investigation suggests that efficacious financial management should not be conceived as only a mere consequence of knowledge and confidence to use it, but rather as the outcome of the joint influence of cognitive aspects and social influences that affect individuals. In fact, in the present work, the impact of investment literacy on financial management behavior is explained by the use of investment advices provided, in a social communication exchange, by a financially expert advisor. Therefore, the present study has focused on facets predominantly studied in current economic psychology (Webley et al., 2002).

Following the growing number of works suggesting that personality traits affect financial behavior beyond the influence of people’s knowledge and external factors (Norvilitis et al., 2006; Warmoth et al., 2016), this work shows that NCC plays a moderating role in the relation between investment literacy and financial management behavior, mediated by investment advice use. Thus, our evidence shows how the personal tendencies of seizing and freezing influence predictors of financial management behavior. On this regard, results show a two side picture. From one side, as we expected, seizing is negatively related to financial behavior; which suggests that individuals with higher tendency to reach quickly a knowledge, a solution to some financial problem, the lower the rate of financial practices. On the other side, contrary to our expectations, individuals that look for financial advice and with high NCC, both for seizing a solution and for freezing it, probably accept quickly the suggestion from the advisor and start to implement it consistently and repeatedly, thus improving their financial performance, in comparison to individuals with lower NCC that may take longer to implement the advice provided by the financial advisor.

This work presents a new viewpoint of how to improve financial behavior among youth and, therefore, can contribute to increasing the efficacy of early interventions to develop responsible financial behavior (Gariepy et al., 2017). Firstly, confirming previous studies (e.g., Calcagno and Monticone, 2011; Collins, 2012), it seems that to benefit of financial advice it is, at least, useful (if not, necessary), to have a good level of financial literacy. Thus, educational, social and political systems should consider how to create opportunities for young adolescents to experience and practice financial competences. Secondly, in this same line, intervention strategies should be oriented toward increasing the coherence between knowledge, expert advice, and financial management behaviors to practice the specific behaviors of saving and investment during young adulthood. Translating this into concrete practices, early assessment of people’s tendencies of Seizing and Freezing could help to recognize these early propensities and their potential bias in the processing of financial information. For example, special attention should be paid during adolescence to these psychological traits to help people develop strategies that compensate these tendencies and reduce their potential negative impact on processes of making complex decisions which may require more time for the analysis and processing of more complex information (Gerlach, 2017). Following these recommendations, parents and educators can develop training programs specifically designed to offset those biases.

Thirdly, while the relationship between investment advice use and financial management behavior is not questionable, the present findings indicate that the quality and quantity of the effects are influenced by employees’ NCC tendencies. According to the present findings, financial advisors might rely upon a complementary tool to increase the efficacy of their interventions. In particular, by monitoring the level of NCC of investors, they may provide some customized services. This would support the idea that not all the products or services fit all the customers, but rather that professionals should fine tune their work in relation to investors’ need to remain open or to close and fix the financial suggestions that are provided. If high NCC individuals might be efficient in implementing easily and quickly the advices provided to them, it is also necessary to remind them of the need to continue to search regularly the advices, to update, and modify financial choices that might become outdated and no more matching the financial situation of the market. In comparison, they must present much wider and more complex financial solutions to low NCC investors, to satisfy their need for extended information processing and thus, facilitate their passage to the actual and concrete financial behavior.

This study presents some limitations that should be considered. Firstly, even though we have considered some cognitive, social, and personality variables in accordance with Huston (2010) model, many other variables could have been considered and should be considered in future research. When referring to long-term economic planning, young workers’ expectations about occupational security, career development, promotion, and progress might also influence their financial management behavior (Ekici and Koydemir, 2016).

Secondly, in this study we measured financial management behavior by tapping participants’ perceptions of their behavior; future studies should include real daily behaviors (e.g., checking one’s bank account, making a monthly budget, controlling credit card expenditures), for example, using research procedures like day reconstruction methods or experience sampling.

Thirdly, in this study we used a 3 months’ lag time between each wave and the following. This lag time allowed anyway to detect a significant relationship between financial literacy and use of financial advice, and between this latter and financial behavior. However, time between waves might be extended to investigate how long is the effect of financial literacy on investment advice, and especially how long such advices may affect financial performance. Fourthly, another limitation is that investment literacy was included only at a first point in time, precluding the possibility of establishing the reverse causation between behavior and knowledge. A research design including the same three variables in each wave, will allow to investigate if, for instance, it is an underperforming financial situation to stimulate the search of financial advices.

Fifthly, in this study, we did not deal with attitudes toward financial professionals, such as customers’ trust and anxiety when consulting them (Grable et al., 2015). In future studies, one might directly ask participants what they think and feel about their financial advisors and incorporate this information as a moderating variable.

Finally, financial literacy studies in general showed another limitation that is due to the well-known association between lower literacy with poor health, low income, and other undesirable outcomes but, as with the present findings on financial management behavior, there is not enough evidence to support any causal direction (Ma, 2016). To date, little is known about the causes and correlates of wrong financial decisions during the life course (Budowski et al., 2016). This kind of knowledge needs to be improved, despite the difficulty of obtaining information from the participants regarding their wealth, financial literacy, and consumer behaviors, and this study does not escape to similar challenges and gaps in data (Manske et al., 2016).

However, this investigation can provide some suggestions to guide future research. First, although we did not examine the impact of gender on financial literacy and financial behavior, it seems that gender differences are related to the quality of financial decisions, even though women’s levels of financial literacy and economic income have improved regarding past decades (Heilman and Kusev, 2017). Therefore, investigating the relationship between gender and NCC could help educators in general, and financial advisors, to design intervention strategies to help women to achieve efficacious financial management (Rudzinska-Wojciechowska, 2017).

Second, research seems to indicate that NCC and risk intolerance are associated. Specifically, risk intolerance is a widely studied variable in the financial setting, but the antecedents of intolerance of risk and ambiguity are still unclear. Therefore, a possible link with NCC could be analyzed, as has been shown in an experimental study (Vermeir and van Kenhove, 2005).

Third, research indicates that executive functions such as impulse control, attention regulation or mental flexibility could be linked to NCC (Dolinski et al., 2016) and to performance in complex tasks and financial well-being. However, recent studies related to the executive functions show that they develop throughout adolescence. Accordingly, early intervention with youth could contribute to improving these cognitive functions, with their consequent influence on NCC and subsequent benefit for the management of complex behaviors, like finances (Barnhoorn et al., 2016; Urquijo et al., 2016).

Lastly, NCC and its correlates of ambiguity intolerance and risk aversion have always been analyzed from an individual perspective. However, recent works propose the possible influence of social comparison in decision making in general and, specifically, in risk-taking behavior (Wang et al., 2016). In this sense, it would be interesting to analyze in future works the influence of the social gains of decisions and their possible interaction with the decision-makers’ NCC.

Financial literacy and decision making should be further explored to better understand how health and well-being are influenced by them during the life course. This research could help societies and policy makers to reduce the considerable economic and public health challenge that posed fast population aging, associated with low financial knowledge and overconfident decision making (Khan et al., 2016). Ultimately, such data will guide interventions to improve literacy and promote independence, wealth, health, and well-being among people from young adulthood to old age.

## Author Contributions

GT, MH-S, and SZ designed the research, analyzed the data, and wrote and revised the manuscript. GT collected the data.

## Conflict of Interest Statement

The authors declare that the research was conducted in the absence of any commercial or financial relationships that could be construed as a potential conflict of interest.
